# Maternal behaviour in domestic dogs

**DOI:** 10.1080/23144599.2019.1641899

**Published:** 2019-07-21

**Authors:** Karina Lezama-García, Chiara Mariti, Daniel Mota-Rojas, Julio Martínez-Burnes, Hugo Barrios-García, Angelo Gazzano

**Affiliations:** a Neurophysiology, Behavior and Assessment of Welfare in Domestic Animals, Department of Animal Production and Agriculture, Universidad Autónoma Metropolitana (UAM), Mexico City, Mexico; b Department of Veterinary Sciences, University of Pisa, Pisa, Italy; c Graduate and Research Department, Facultad de Medicina Veterinaria y Zootecnia, Universidad Autónoma de Tamaulipas, Victoria, Mexico

**Keywords:** Maternal care, dog behaviour, whelping, nursing, puppy welfare

## Abstract

Mammalian parental care, in most of the cases, is given by the female, who provides food, warmth, and protection. In domestic dogs, maternal behaviour shown by the dam mainly consists of contact, nursing, grooming/licking, play, punishment, thermoregulation, and motion. Peer-reviewed literature published between 1952 and 2018 was retrieved from CAB Abstracts, PubMed, ISI Web of Knowledge, Scopus and book chapters. Keywords for this search included the following terms: behaviour, bonding, altricial, precocial, offspring, maternal, whelping, nursing, domestic dogs, female dog, aggression, puppies, anogenital licking. In this review, we reported and discussed scientific information about maternal behaviour in domestic bitches, comparing altricial vs precocial species; the importance of the bonding, grooming/licking and nursing, and their impacts on puppies’ behaviour; altered maternal behaviours such as aggression, cannibalism, rejection, and also the relation between hormones and maternal care behaviours. We concluded that the level of interactions between the dam and the puppies influences the physiological, cognitive and behavioural development of the litter, and the main hormones in the bitch for inducing maternal care behaviours are estradiol, oxytocin, prolactin and progesterone.

## 1. Introduction

Mammalian parental care in dogs is mainly performed by the dam, who provides food, warmth [1,2], shelter and protection from predators and conspecifics [3,4], helping to the offspring´s survival and their mental and physical well-being [5,6]. It has been documented that maternal care has an impact on individuals’ development in many species, including rodents, pigs, non-human primates, humans and recently in adult dogs [7–9]. The quantity of the relationship between the dam and the offspring interpose on physiological, cognitive and behavioural offspring’s development [10]. The maternal behaviour detected on bitches towards the litter includes contact, nursing, licking (mainly anogenital licking, that allows newborn puppies to urinate and defecate in a duly manner), play, punishment, thermoregulation and motion [11,12].

Maternal behaviour, delivery and nursing synchronisation assure that the dam responds to offspring’s needs in time. A variety of studies in newborn mammals prove that maternal behaviour directly impacts on the neonatal survival [13–15] since mortality rates between birth and weaning are related to maternal and neonatal behaviour [16–21].

Dogs are altricial animals and, for that reason, the establishment of dam-offspring bonds can take days or even weeks to develop. Nevertheless, nest building, licking, nursing and puppy’s rearing is present in most of the cases. In comparison with wild canine species, domestic dogs are considered to have a reduced disposition for maternal behaviour [22], and therefore, human intervention is sometimes required to increase puppies’ survival [12].

This review aims at discussing and comparing provided data by scientific papers about domestic dogs maternal behaviour. Throughout this paper, we will address several issues such as differences in maternal behaviour between both altricial and precocial species, maternal skills, licking and dam-offspring bond significance and its impact on long-term puppies’ behaviour, abnormal maternal behaviour, and the relationship between maternal behaviour and bitches’ hormonal levels. In this way, current findings allow readers to get a comparison between the results of different authors’ studies, and also to have a conclusion about the last published data on bitch maternal behaviour.

## 2. Maternal behaviour in altricial vs precocial species

Natal viability is associated with foetal maturity, environmental conditions, and maternal care [23]. Usually, altricial and precocial terms are used to indicate an early for late stage of development at birth in mammals and birds. The altricial-precocial scale describes the degree of behavioural and morphological maturation of the offspring at birth or hatching [24]. In precocial species, offspring require minimal parental care and are relatively mature, able to move and can feed themselves (precocial birds) or forage independently from the start while still being nursed (precocial mammals). In contrast, altricial canids, rodents, felids are initially incapable of moving around on their own and require extensive parental care, like brooding or food access [25]. In altricial species, such as the dog, offspring are unable to care for themselves at birth, are usually born deaf and blind, and have limited movement [12,26].

The ability of the newborn to survive and to grow within the born environment depends on the vital organs’ maturation level at birth. Organs of placental altricial neonates are capable of maintaining its vital functions and the short-term metabolic changes [27,28]. However, in some species, neonates need a dam to hatch them or to feed them [25]. Altricial species dams (as rodents, canines, and felines) built a nest where they give birth to their offspring, which are not fully developed and have limited sensorial and motion skills [4,29]. Maternal behaviour largely differs across species. For instance, once the rodent dam has gathered its offspring within the nest, it spends most of the time in a nursing position, grooming them and eliminating faeces and urine [30]. In rabbits, maternal behaviour is different: dam only suckles rabbits once or twice a day, for periods no longer than 10 minutes each [31]. Primate dams instead carry offspring most of the time and hold it with its strong hands [32]. Dogs are born blind and deaf, which limits newborn care [26]; for that reason, puppies’ physical and social development is mainly determined by the interaction with the dam [33]. During the fifth week postpartum, offspring frequently gets out of the nest, and weaning starts [34].

In precocial species (most of the ungulates), offspring requires less parental care, as soon after birth they can move and can graze between breast-feedings [25]. Precocial placental newborns show an organs’ advanced development state to keep vital functions, thermoregulation, and a highly energetic performance [27]. Immediately after birth, dam licks offspring until they are free of amniotic fluid and placental remains [35]. Precocial species deliver small litters, with fully developed offspring, capable of following the dam immediately after birth. Within these species, dams develop discriminatory maternal care that allows only its offspring to suck and avoid other animals to do so [4,18].

The bitch nurses the puppies intensively during the first days, and hardly leaves the nest. If the puppies roam around, the dam will lick them to return them to the nest. From birth to three or four weeks old, the dam needs to stimulate urination and defecation (through anogenital licking), and she will eat any waste, feed the puppies, and provide a heat source to maintain them at stable body temperature [36,37].

All the reviewed data agree that maternal behaviour shows meaningful differences between precocial and altricial species, which is less intense in precocial species than in altricial ones. For a more comprehensive review on altricial and precocial social complexity, see Scheiber et al. [25].

## 3. Maternal behaviour and hormonal influence

In all mammal species, the period surrounding the birth is characterised by an increase of plasma estradiol, prolactin, and cortisol levels, and by the activation of the oxytocinergic system at the time of delivery [4].

The factors causing the onset of typical maternal behaviours are still subject to debate and widely studied in rodents [38–41]. In rodents, the existence of an attraction-acceptance neural circuit, that would inhibit the activity of another defensive antisocial neural circuit, has been hypothesised [38].

The hormonal constellation present at the end of pregnancy allows a rapid display of maternal behaviour and acceptance of newborns, activating the attraction-acceptance circuit [42].

Female primiparous rats immediately demonstrate maternal behaviour, while virgin female rats when exposed for the first time to newborns, show avoidance lasting for 3–4 days [43].

On one hand, in pregnant rats, the increase in the blood concentration of oestrogens and prolactin would activate the medial preoptic area (MPOA) of the hypothalamus, making it capable of responding to stimuli from newborns, inhibiting the antisocial defensive circuit and activating the attraction-acceptance circuit (the mesolimbic dopamine system, MDS), resulting in prosocial maternal responses [44]. On the other hand, recent studies support the hypothesis that, in rats, unfamiliar olfactory stimuli from pups activate the neural circuit responsible for pup avoidance in virgins, composed by the olfactory bulb, medial amygdala, anterior hypothalamic nucleus and periaqueductal gray [43].

According to Numan et al. [43], MPOA neurons, primed by prolactin and estrogen, become responsive to pup stimuli and activate neurons of the ventral tegmental area of the midbrain, where MDS has its origin, and release dopamine in the nucleus accumbens, located in the subcortical cerebral hemispheres. Dopamine causes a reduction in the response of the nucleus accumbens to stimuli originating from the defensive antisocial neural circuit. The nucleus accumbens would also be inhibited in its regulatory activity of the ventral pallidum, a motivational control region. The ventral pallidum could, therefore, become active and allow the appearance of maternal behaviour.

**Figure 1.**
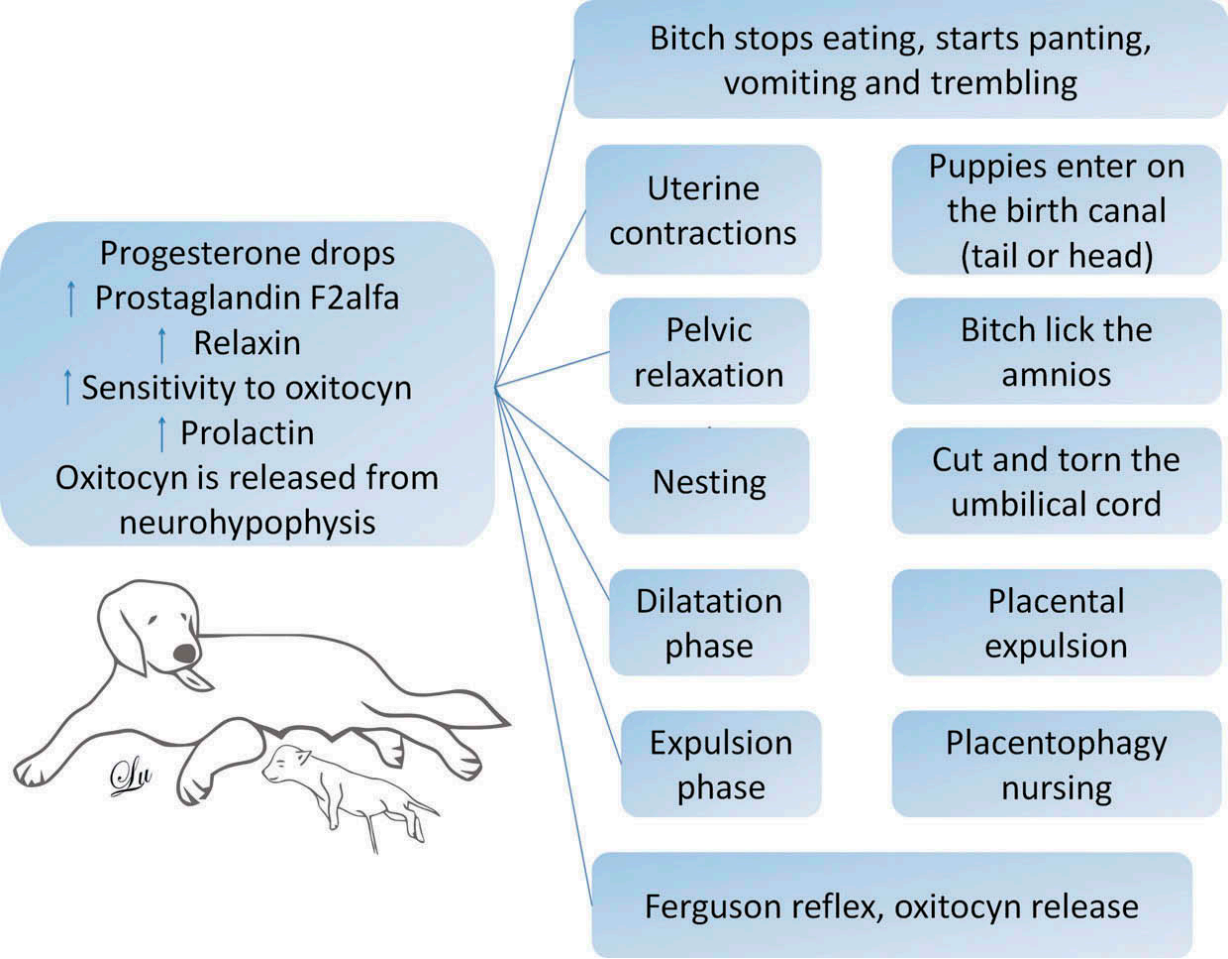
The behavioural repertoire of the bitch at birth. In order to trigger labour, there must be a decrease in the progesterone, followed by the increase of the prostaglandin F_2_, which creates an increase in the oxytocin sensitivity in the bitch’s uterus. At this moment, the dog begins with the construction of the nest or to move away to some dark place. Likewise, it increases the prolactin, and the release of oxytocin from the neurohypophysis, and with signs of fainting, vomiting, inappetence, trembling, and uterine contractions start. Here it is when the dilation phase begins. Subsequently, the expulsion phase and the Ferguson reflex are activated, with more release of oxytocin; uterine contractions emerge, and the fetus places itself, either anteriorly or posteriorly in the birth canal. The bitch starts to lick her vulva and to break the amnion, she cuts and tears the umbilical cord, and subsequently, there is the placental expulsion, the bitch eats the placenta, and because of the relaxin, prolactin, and oxytocin stimulus, the lactation starts.

It is generally accepted that dams identify their offspring by olfaction and this seems related to oxytocin release [9,18,20]. Oxytocin, a neuropeptide with the dual action of hormone and neurotransmitter, is another molecule largely involved in triggering maternal behaviour. Oxytocin enhances maternal interest in young dogs by reducing anxiety and stimulating maternal care [42]. The posterior pituitary gland is the site of oxytocin release after neurologic stimulation [45,46]. There are two recognised stimuli to induce oxytocin release. The first is Ferguson´s reflex, that consists of the pressure of the head of a puppy into the cervix during parturition [45,47,48]: the cervicovaginal stimulation during labour and the secretion of prolactin, along with the presence of estradiol and progesterone, release oxytocin by producing reactions in the maternal brain [47] (Figure 1). The second stimulus is the suckling stimuli of the mammary glands by the puppies [45].

Oxytocin is produced by the neurons of the paraventricular nucleus of the hypothalamus, which projects to many regions of the brain, including the MPOA. The action of oxytocin would be that of potentiating the stimulating activity of MPOA on MDS [44].

In rats, the long-term persistence of maternal behaviour, also known as “maternal memory,” is also maintained by oxytocin and dopamine action in the nucleus accumbens shell [49]. Oxytocin is also responsible for the synchronised uterine contractions during parturition and the further dilation of the cervix. In contrast, progesterone maintains pregnancy by promoting the secretion of intrauterine glands to sustenance the fertilised eggs, stimulates the expansion of the mammary glands, and induces maternal behaviour. Further, the decrease of progesterone and a subsequent rise in prolactin cause the common maternal behaviours seen in dogs, including nesting [45,50] (Figure 1).

After birth, puppies are exposed to particular pheromones secreted by the sebaceous glands located in mother intermammary sulcus, the area in between her breast where puppies detect them upon nursing. Because these pheromones provide calm, comfort and a sense of well-being to the puppies, they are called Dog Appeasing Pheromones (DAP) [46,51,52].

Another factor related to the duration of the elevation of some hormones is the parity number by which the bitch is passing; that is, it can be different in primiparous and multiparous. In a study performed by Seki et al. [53] the initial increase of progesterone levels was of shorter duration in primiparous bitches [4].

According to the above, we can conclude that the levels of oxytocin, prolactin, and progesterone are closely related to maternal behaviour. Oxytocin mediates several forms of affiliative behaviours, including parental care, and grooming [54–56] the formation of a pair-bond, as well as the establishment of the relationship between dams and offspring [57].

A study using potent prolactin inhibitors, mostly dopamine agonists confirmed the role of prolactin as luteotropic hormone from day 30 of pregnancy and beyond and that it is crucial for the preparation, initiation and sustaining lactation, as well as for the activation of maternal and sexual behaviour [58]. Prolactin seems to be involved in ensuring maternal behaviour, including the preparation for delivery and the care of the litter, although it is not yet clear how it shares these effects with oxytocin [59].

**Figure 2.**
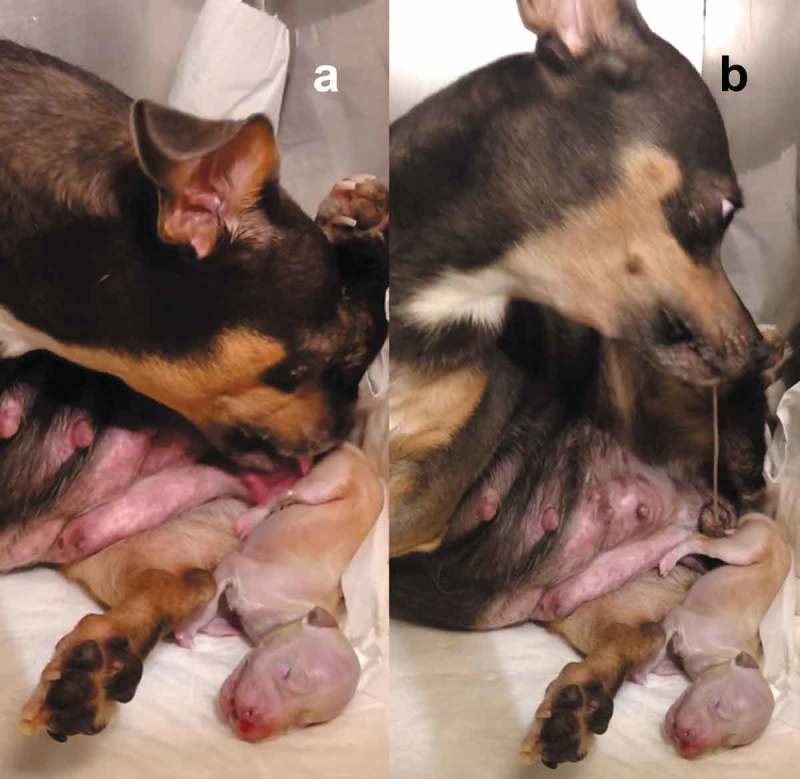
(a) The bitch breaks the amnion and devours the fetal membranes, initially from the puppy’s head so it can breathe quickly, and then cleans the rest of the body, licking it and in the same way stimulating the offspring (Photo MVZ David Rivera). (b) The bitch tears the umbilical cord keeps licking and cleaning the puppy, and when the dam throws the placenta, she starts eating it to avoid the nest contamination and thus not attract predators. Placentophagy favours the uterine involution and milk production.

During the birthing process, the bitch licks the placenta and start eating the foetal membranes, the remains of the placenta and also tears the umbilical cord [36,45,60–62], reducing the contamination of the nest and avoiding predators [36] (Figure 2(a,b)). Cleaning the newborn, and consuming the amniotic fluid and placenta, are common behaviours within mammals [63], except the big aquatic mammals (cetacean) or semi-aquatic (pinniped); likewise, the dams produce vocalisations to keep close the offspring and drive away from the predators [4].

In most bitches and queens, a typical maternal behaviour is that they wait until the whole litter is born to feed them. It is generally accepted that dams identify their offspring by olfaction, and this seems related to oxytocin release [45,60].

**Figure 3.**
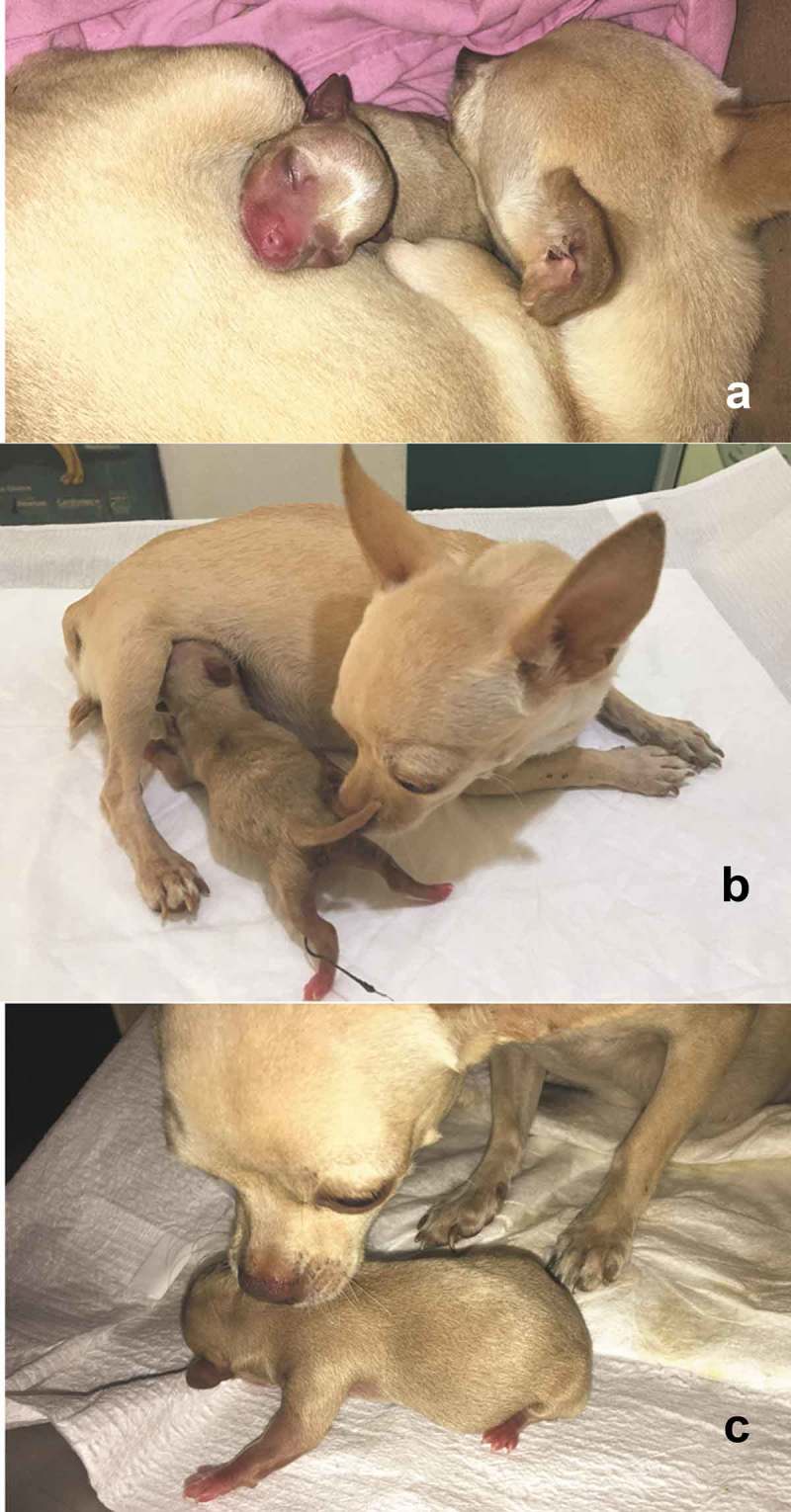
Maternal behaviour at parturition. (a) The bitch, provides warmth and protection to the puppy and stays with it most of the time. It only leaves the nest to feed, urinate and defecate. The rest of the time it is taking care of the offspring at least during the first three weeks of the puppy’s life. (b) The bitch pushes out the puppy with the snout to mammary glands to facilitate its nursing. By doing so, it takes advantage of licking the offspring’s genitals to stimulate the puppy to urinate and defecate. In this picture, we can observe the offspring’s umbilical cord, which will detach approximately on the third day of birth. (c) Since the puppy fails to move around by itself, bitch helps it by gently grabbing the neck with the muzzle to move it from place to place.

The first contact of puppies and kittens with the mother’s tit is by the sense of smell and by trials and errors. The dam uses its paws to push the newborns towards the nipples and allow them to nurse. Also, the puppies and kittens press the mammary gland to stimulate the descent of the milk [45]. (Figure 3(a,b,c)).

After feeding the offspring, the dam stimulates in them urination and defecation through anogenital licking.

Those mentioned above continue when the puppies and kittens start moving and until three weeks of age.

During the first week after parturition, the dam rarely leaves alone the offspring, only to eat, urinate and defecate. Maternal care can differ depending on the experience of the mother: maternal care (specifically licking the anogenital area, lactation and staying in contact with the puppies) during the first three weeks is markedly higher in primiparous than in multiparous mothers [64]. Later (the 4^th^ week after delivery) the dam becomes evasive, rejecting the offspring to suckle as their teeth start to erupt, and leave the litter for extended periods [45,60]. In primiparous dams, a higher licking of the anogenital area, nursing and contact with the puppies from day 1 to 21 was found, which led to a more significant amount of maternal care in the third week compared to multiparous mothers.

### 3.1. Milk production from the hormonal point of view

The study of the biological mechanisms that influence on regulates pregnancy, delivery, and lactation, is very important to understand maternal responses. Maternal behaviours usually arise immediately after birth, where the female quickly shows great interest in the newborn. However, the most essential and natural maternal behaviour in mammals is lactation (Figure 5(a,b)), which occurs right after birth and ensures that newborns grow up healthy and strong [4].

**Figure 4.**
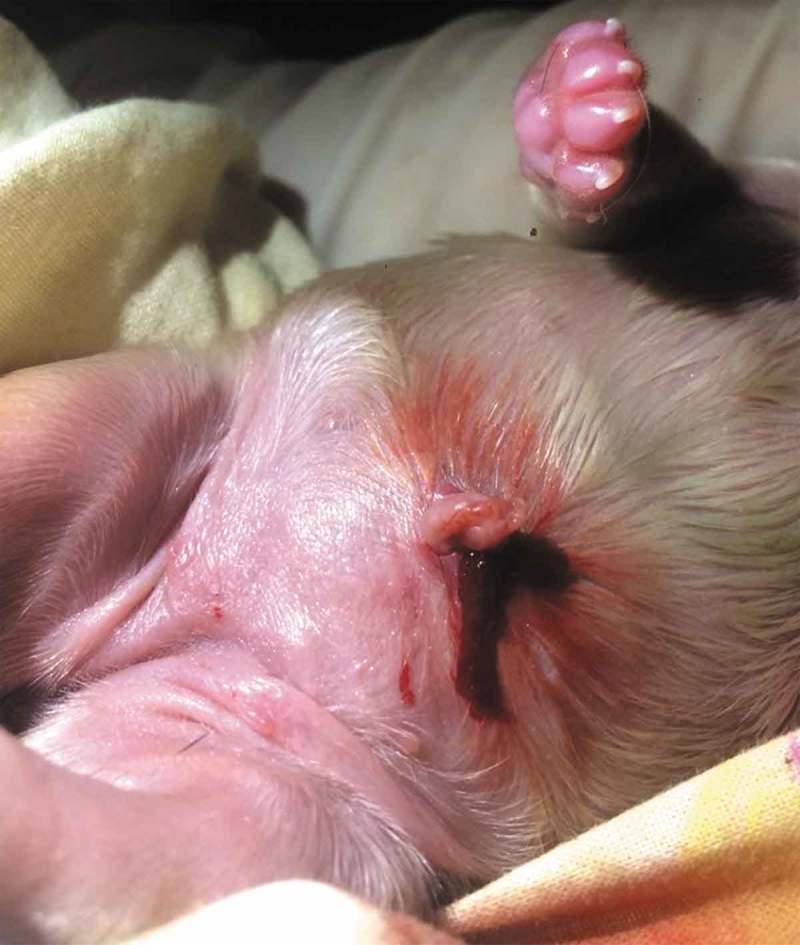
Sometimes due to inexperience (primiparous) or nervousness, the bitch pulls too much the umbilical cord of the newborn when trying to tear it and this may cause injuries in the puppy (such as evisceration) or inclusive cannibalism (Photo MVZ Gibran Olivera Rodríguez).

**Figure 5.**
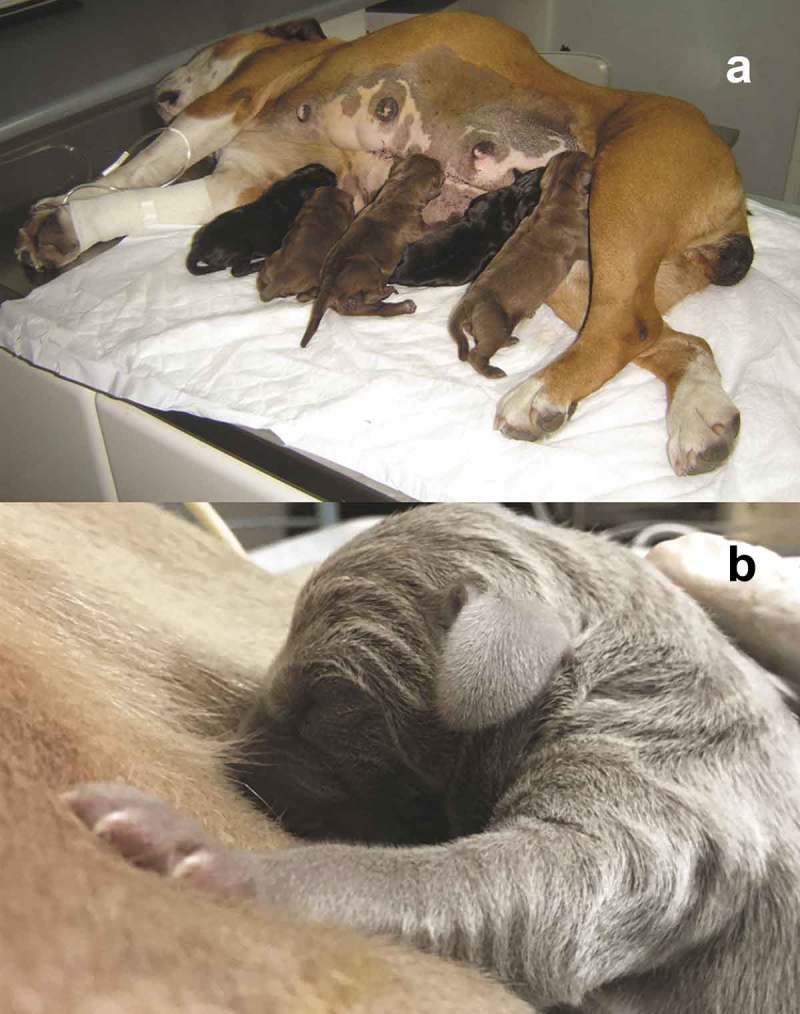
(a) The bitch acquires a lateral decubitus position to nurse the offspring; they look for the closest nipple and begin to suck hard. (Photo MVZ Esp. Juan Jose Santiago Garcia). (b) The puppy indistinctly selects a nipple and begins to suck and make massage movements that stimulate the mammary gland to release milk (Photo MVZ Esp. Juan José Santiago García).

The hormones prolactin and oxytocin play an essential role during lactation. Prolactin promotes milk production and incites normal maternal behaviour. Prolactin release is stimulated during pregnancy, and its blood levels rise suddenly at parturition, while other hormone concentrations abruptly decrease [45]. Oxytocin stimulates descent of milk into the mammary glands, and its blood levels are high during nursing. A correlation between the quantity of oxytocin secreted with the efficiency of milk production is described in some species [50].

## 4. Offspring grooming

Maternal licking in rats is associated with changes in offspring behaviour, such as maternal and anxiety-like behaviour [65]. It also affects receptors of glucocorticoid [66], oxytocin, vasopressin [67] and estrogen [68].

Published studies in animal models remark the relevance of the extent of nursing, anogenital and body licking, and the close body contact of the dam with the offspring. These behaviours may further influence the emotionally [69] and stress response and social skills of the neonates [68,70,71]. Moreover, Champagne et al. [68] describe that individual pups within a litter receive the same amount of licking and grooming.

For all the above, it is unquestionable that licking is one of the leading maternal behaviours that may further affect the puppies’ behaviours and the interactions in their environments.

## 5. Bonding between dam-offspring and maternal care

For parents, recognising their offspring help to prevent misdirected parental care, limits energy losses, and ensures reproductive success. On the other hand: for young animals, recognition of parents is also essential to their survival, since, in most species, parents feed only their newborns. There is evidence that parent-offspring recognition is crucial for colonial species, though the degree of recognition seems to vary among different species depending to some degree on environmental constraints [4,72]. For the offspring of all mammal species, their mothers are the essential social contact during the first months of life as they facilitate acquiring information about physical and social environments [73].

Besides providing genetic material, parents also play a fundamental role in the offspring development [33]. The quality and quantity of the relationships between the dam and the offspring take part in the puppies’ physiological, cognitive and behavioural development [10,70,74]. The maternal behaviour towards the offspring during the early stages plays an essential role in their development, just as the following character and behaviour. For example, maternal behaviour alters the hypothalamic-pituitary-adrenal axis or stress responsiveness pathways of the neonate, and these changes persist throughout adult life [12]. In two studies conducted by Guardini et al. [10,75], it has been found an association between the amount of maternal care provided by the mother to each puppy and their behaviour at two months of age, suggesting that the extent of maternal care that puppies receive during the neonatal period influences their coping strategies at two months of age. However, the patterns of behavioural responses widely differed according to the environment in which puppies were raised, so that authors suggested that maternal care contributes to the adaptation of puppies to their environment, especially to the social relationship with humans [75]. Besides, a high level of maternal care in dogs influences physical and social engagement, aggression, and lower levels of anxiety and fear [8,10,76].

In the same way, in a study accomplished by Foyer et al. [8], they taped 22 German shepherd litters during the first three weeks postpartum, to evaluate maternal care variations and their effects on behaviour and temperament in puppies around one and a half years of age. They observed that the maternal care level varied between different bitches, during the first three weeks after birth, and this affected the social and physical engagements, as well as aggression, in the offspring when they became adults. Foyer at al [8]. also found that the amount and quality of maternal care would vary during the suckling period and that better maternal care would lead to less reactive, more confident and explorative puppies.

The maternal care and the early postnatal environment may also have marked effects on subsequent stress-related behaviours. Tiira and Lohi [76] described the correlation between maternal care in puppies and the later development of anxiety. They collected data from a family dog population (n = 3264) to evaluate the association between environmental factors and fearfulness, noise sensitivity, and separation anxiety. The study revealed that fearful dogs had had significantly fewer socialisation experiences and lower quality of maternal care during puppyhood. Noise sensitivity and separation anxiety was associated with a lower level of daily exercise. Authors suggest that dogs share many of the same environmental factors that contribute to anxiety in other species, such as humans and rodents.

Nonetheless, there are limited studies about maternal care as a predisposing factor for anxiety, because it is a relatively new concept in dogs, but described in other altricial species [12]. Also, the environment may affect puppies’ behaviours once they are adults. A study conducted by Appleby et al. [77], described higher aggression ranges in adult dogs when they were puppies raised in non-domestic maternal environments, compared to those raised in domestic maternal conditions.

A study conducted by Bray et al. [78], follow 98 puppies from birth to adulthood, who were allocated to be guide dogs and they observed a link between high levels of maternal behaviour and a higher probability of program failure. Also, that the dams who require a higher effort from puppies to accomplish nursing are more likely to have successful offspring to be guide dogs; on the other hand, dams whose lactation requires less effort from puppies are more likely to have offspring who will not perform as guide dogs. The same study describes in the temperament tests that there is an association between maternal behaviours and the young adult behaviour, on the same way that Foyer et al. [8] and Guardini et al. [10]. However, unlike them, Bray et al. [78] found that the extreme maternal care is directly linked to adverse anxiety behaviours in dogs when they are young adults, as well as poor problem resolution and the decrease of latency at the first barking period.

Another study conducted by Bray et al. [79], monitored maternal interactions in twenty-one litters from three different breeds. The study revealed that a mother’s attitude and actions toward her offspring varied naturally between individuals and that these variations could be summarised by a single principal component, which they described as Maternal Behaviour. This element was stable across weeks, associated with the breed, litter size, and parity, but not redundant with these attributes.

## 6. Abnormal maternal behaviours; aggression, cannibalism, reject, pseudocyesis

Studies have identified different causes for abnormal maternal behaviour, such as high prepartum and postpartum stress levels [80–83], hereditary predisposition [84], low serotonin levels [85], and low oxytocin levels [86].

### 6.1. Aggression

Maternal aggression is usually temporal, and it will decrease just as puppies grow up [87]. The aggression is towards whoever approaches the nest, to the puppies or the objects the bitch perceives as puppies during the false gestation [88].

In bitches, aggression may exacerbate by vocalisation of puppies. However, the aggressiveness may be associated with pain, for example, in cases of mastitis [45]. There is a critical role for oxytocin in the onset between the dam and offspring bonding, associated with low stress and fear [89,90], and on the increasing maternal aggression toward threats [91].

The bitches with maternal aggressiveness monitor the nest, the puppies, or when a person or an animal is near. The puppies represent a precious resource for the bitch, in which it has already invested much energy required for a satisfactory pregnancy. The hormonal changes associated with lactation can alter the dam evaluation or perception of the range of resources, and involve protection even in ordinary objects, with an apparent disproportional ferocity. Fears may follow attacks or bites in some cases [88]. The lactating bitches may become aggressive towards humans or even canines. This behaviour may cause difficulties when trying to control the puppies progress and to secure adequate socialisation. The aggression may be accelerated because of environment troubles, especially in anxious or immature bitches, within an unstable social environment [36], and it decreases after a few days or weeks, when the puppies grow up and become less dependent on the dam and, therefore, their defence needs are reduced [88].

### 6.2. Cannibalism

Maternal cannibalism is also called cronyism [45], and it is a condition where a dam consumes her offspring after killing them [92,93]. It is abnormal maternal behaviour unless it is a strategy to reduce litter size, balance the sex ratio of the offspring, eliminate the offspring that has come out defective [80,94] or when there is a poor significant environmental condition [95]. The dam can accidentally kill and devour a puppy during the process of cutting the umbilical cord (Figure 4) [36]. Although some biological factors, such as low levels of oxytocin and blood lipids are related to a failure in maternal behaviour in different animals, this had not been investigated in detail. Kockaya et al. [93] measured these substances in the serum of 15 adult Kangal bitches, who had a cannibalism background, compared with 15 samples of bitches from the same breed, who had no maternal cannibalism background. The results revealed significantly lower levels of these substances in bitches with a maternal aggressiveness background. The oxytocin levels of 3.58 + 0.43 ng/ml and cholesterol at 125.50 + 8.6 mg/dL in dogs with cannibalism, compared with oxytocin of 9.68 + 1.58 ng/ml and cholesterol at 159.18 + 13.85 mg/dL in bitches with normal postpartum behaviour. Thus, they concluded that oxytocin is an essential neuroendocrine factor in bitches, to develop normal maternal behaviour.

Bitches rarely direct severe aggression towards their puppies and are more likely to manifest such behaviours with their first litter. However, there are some cases in which bitches have caused severe injuries and are considered a hereditary trait [96]. Causes of cannibalism include pain (usually associated with mastitis) and eclampsia, a large litter, stress, and overcrowding [45].

### 6.3. Rejection of the offspring

Rejection of the offspring or maternal negligence, it is uncommon, but some bitches may fail to attend their puppies regarding warm, nutrition and urine and faeces elimination [97]. Primiparous dams are more prone to rejection. Also, in anxious dams who leave the nest frequently, those with premature litters (less than 57 days of gestation), litters born through a caesarean section or where there are many discomforts in the environment [36]. Breeding practices can also alter the behaviour of the bitch towards her litter, such as removing the pups from the nest areas and holding them for a short period [98].

Rejection behaviour of the offspring is described in bitches under some conditions, including when one or two puppies are repeatedly moved from the nest or hidden, then the dam perceives that something is wrong with that puppy. Also, bitches routinely reject pups that are cold or are not moving. However, if the entire litter is rejected, it suggests that something is wrong with the dam and medical assistance is necessary to identify clinical signs of morbidities as mastitis, metritis, and eclampsia, or stressing factors (overcrowding, large litters). Some primiparous bitches are poor mothers, especially if they are nervous or anxious. However, many of these dams tend to be good mothers with subsequent litters [45].

### 6.4. Pseudocyesis

Pseudocyesis (also called false pregnancy) is a common condition where the non-pregnant bitch goes through the same hormonal changes as the pregnant one. It can depend on both serum prolactin levels and the sensitivity of tissues to this hormone [36]. Pseudocyesis can be underdiagnosed in bitches and may be the cause of some cases of behavioural problems, including aggression [99]. At the end of dioestrus, when progesterone concentrations fall, and prolactin concentrations rise, the female dog exhibits mammary development, produces milk and may show behavioural changes as if she is whelped such as nesting and mothering of inanimate objects and some bitches may become aggressive [45]. Apart from typical maternal behaviours such as care, grooming, vomiting, the dam may show the enlargement of the mammary glands, as well as restlessness and construction behaviour nest, similar to the pregnant bitches that have delivered – before, anxiety, agitation, lethargy, aggression and pain [36,88,99–101]. Roth et al. [99] developed a survey applied to 2000 veterinarians, to know the frequency of cases of pseudocyesis in dogs and the signs observed. The results revealed that 96% of the respondents reported having observed cases of pseudocyesis with changes in maternal behavioural (the most common was that the bitches adopted objects as if they were puppies). Pseudocyesis was reported in 49% in sterilised bitches, and the most common physical sign was the enlargement of the mammary gland and milk production (89%). Besides, 97% of veterinarians reported cases of aggression associated with pseudocyesis.

## 7. Conclusion

Dogs are altricial species that depend entirely on the dam to survive, obtaining warm, food, movement, protection from predators and stimulation through anogenital licking to eliminate waste. The main difference in maternal behaviour between altricial and precocial species is that, in the first, the dam-breeding link takes from one day until weeks to be established, while in the precocial, this bond is generated even in hours. For delivery and maternal behaviour to take place, a set of factors must happen both in the foetus and in the bitch, thus activating the series of hormones involved, such as oestradiol, cortisol, progesterone, prolactin, oxytocin, and relaxin. The normal maternal behaviours include the creation of the nest, contact, nursing, licking, play, punishment and within abnormal maternal behaviours authors describe aggression, pseudocyesis, cannibalism, and abandonment of the offspring. Abnormal maternal behaviour in the bitch is related to low levels of some hormones, mainly oxytocin. All the authors revised agree on the relationship between maternal care of breeding and the development of desirable or undesirable behaviours in puppies when they are adults. With more maternal care, the puppy becomes more dependent, anxious and has a harder time adapting to its environment. However, to date, few studies describe maternal behaviour in domestic dogs, so it is necessary to continue researching this topic.
